# A literature review of the disruptive effects of user fee exemption policies on health systems

**DOI:** 10.1186/1471-2458-12-289

**Published:** 2012-06-08

**Authors:** Valéry Ridde, Emilie Robert, Bruno Meessen

**Affiliations:** 1 Department of Preventive and Social Medicine, Medical Faculty, University of Montréal, 3875, rue Saint-Urbain, Montréal, QC, Canada; 2 Centre de recherche du Centre hospitalier de l’Université de Montréal, Montréal (CRCHUM), Montréal, Canada; 3 Institute for Tropical Medicine, Antwerp, Belgium

## Abstract

**Background:**

Several low- and middle-income countries have exempted patients from user fees in certain categories of population or of services. These exemptions are very effective in lifting part of the financial barrier to access to services, but they have been organized within unstable health systems where there are sometimes numerous dysfunctions. The objective of this article is to bring to light the disruptions triggered by exemption policies in health systems of low- and middle-income countries.

**Methods:**

Scoping review of 23 scientific articles. The data were synthesized according to the six essential functions of health systems.

**Results:**

The disruptions included specifically: 1) immediate and significant increases in service utilization; 2) perceived heavier workloads for health workers, feelings of being exploited and overworked, and decline in morale; 3) lack of information about free services provided and their reimbursement; 4) unavailability of drugs and delays in the distribution of consumables; 5) unpredictable and insufficient funding, revenue losses for health centres, reimbursement delays; 6) the multiplicity of actors and the difficulty of identifying who is responsible (‘no blame’ game), and deficiencies in planning and communication.

**Conclusions:**

These disruptive elements give us an idea of what is to be expected if exemption policies do not put in place all the required conditions in terms of preparation, planning and complementary measures. There is a lack of knowledge on the effects of exemptions on all the functions of health systems because so few studies have been carried out from this perspective.

## Background

If illness constitutes a major shock for households, the cost of healthcare is a second shock that strikes harshly and can drag them into poverty [1]. In Burkina Faso, 80% of poor households go into debt or sell assets to pay healthcare costs [2]. User fees in public services contribute to these catastrophic expenses [3], reduce health services utilization [4,5] and are a major impediment to universal healthcare coverage [6,7]. One solution being considered to improve access to care is to make services free at the point of service. Many countries, such as South Africa, Mali, Niger and Ghana, have therefore implemented exemption policies, sometimes targeted to population groups (e.g. children under the age of five years) or to specific services (e.g. caesareans).

Among the many articles published on this topic, two knowledge syntheses have been carried out. One presented the available scientific knowledge on the emergence, formulation, implementation and effects of exemption policies in five African countries and identified research needs [8]. The other was a systematic review aimed at evaluating the effectiveness of funding mechanisms to promote healthcare access for the poorest, including user fee exemptions [9]. Despite the poor quality of their research design, the authors concluded that the studies had shown that exemptions had a positive effect on health services utilization, but that they were not sufficiently effective to protect the poorest.

Even though these policies have produced an increase in the demand for services and therefore would appear very technically relevant, they raised a number of pragmatic concerns. In fact, these reforms have often been organized within unstable health systems that are not much used by the population and whose organizational deficiencies and dysfunctionalities are well known [10,11]. While still subscribing to the principle of point-of-service fee exemption, decision-makers from 15 African countries reiterated these concerns at a meeting held at the end of 2010 [12].

A health system is a collection of services, persons and actions whose interactions are complex and interrelated. As has been affirmed by the World Health Organization (WHO) and others before [13], for a health system to be effective and equitable, its different functions must work in unison, and any change will therefore have an effect on each function [14]. Thus, exemption can modify a health system’s equilibrium and disrupt its essential functions. The objective of this article is to identify the disruptions in health systems that are caused by these new public policies.

## Methods

Our analysis was based on a survey of the scientific literature [15]. That survey took the form of a scoping study, an approach which summarizes all scientific knowledge on a specific topic, regardless of the type or quality of the studies’ designs. This summary is useful to better understand the nature of the scientific studies currently being done and to identify possible shortcomings. It differs from, and is often seen as a precursor to, systematic reviews that synthesize the most robust scientific data by using methods that limit biases and random errors [16]. We applied the systematization of the scoping study process proposed by Arskey and O’Malley [17]. Details of the documentary research strategy have been described elsewhere [15].

An article was retained if it: 1) dealt with a healthcare user fee exemption experience on a national scale (public policies); 2) reported original empirical data; 3) involved African countries; 4) mentioned pressures or disruptions in the health system; 5) was published in a peer-reviewed journal or monograph; 6) was published between 1988 and 2009 inclusively; and 7) was in French or English. The studies’ designs were reported using the classification system of the Mixed Method Appraisal Tool [18], an instrument used to describe the quality of studies, whatever their nature.

We examined the content of the articles to identify the elements that produced bottlenecks or pressures on the health system. WHO has defined a health system based on six essential functions (Table 1). We used this framework to analyze the content of the articles retained.

**Table 1:** The six building blocks of a health system

Health system functions	Definition
(1) Service provision	Good health services are those which deliver effective, safe, quality personal and non-personal health interventions to those who need them, when and where needed, with minimum waste of resources.
(2) Health personnel	A well-performing health workforce is one which works in ways that are responsive, fair and efficient to achieve the best health outcomes possible, given available resources and circumstances.
(3) Health information	A well-functioning health information system is one that ensures the production, analysis, dissemination and use of reliable and timely information on health determinants, health systems performance and health status.
(4) Drugs and vaccines	A well-functioning health system ensures equitable access to essential medical products, vaccines and technologies of assured quality, safety, efficacy and cost-effectiveness, and their scientifically sound and cost-effective use.
(5) Funding	A good health financing system raises adequate funds for health, in ways that ensure people can use needed services, and are protected from financial catastrophe or impoverishment associated with having to pay for them.
(6) Governance and leadership	Leadership and governance involves ensuring strategic policy frameworks exist and are combined with effective oversight, coalition-building, the provision of appropriate regulations and incentives, attention to system-design, and accountability.

Source: WHO [14].

## Results

### Description of the studies identified

Our research approach gathered 23 articles (Table 2) from seven different African countries: Ghana, Kenya, Madagascar, Senegal, South Africa, Tanzania and Uganda. The topic most often studied was service utilization or demand (n = 7), not surprising given that improving utilization is usually the primary objective of exemption policies. Few articles looked at policy implementation (n = 3) or changes in service quality (n = 1). The types of study designs included quantitative non-randomized (n = 12), quantitative descriptive (n = 2), qualitative (n = 3) and mixed (n = 6).

**Table 2:** Description of the articles included in the review (n = 23; 1988 to 2009)

Authors and date	Date	Topic	Qualitative	Quantitative randomized controlled	Quantitative non-randomized	Quantitative descriptive	Mixed methods
**Ghana (n = 4)**							
Penfold et al.	2007	Service utilization			X		
Witter & Adjei	2007	Implementation	X				
Witter, Arhinful, et al.	2007	Implementation	X				
Witter, Kusi, et al.	2007	Effect of exemption on health workers				X	
**Kenya (n = 4)**							
Chuma et al.	2009	Facilites' adherence to exemption					X
Mwabu et al.	1997	Service demand			X		
Mwabu et al.	1995	Service demand, and effect on income and quality			X		
Perkins et al.	2009	Costs of service utilization			X		
**Madagascar (n = 1)**							
Fafchamps et al.	2007	Impact of 3 political periods on the health sector					X
**South Africa (n = 4)**							
Bhayat et al.	2003	Service utilization			X		
Walker et al.	2004	Implementation					X
Wilkinson et al.	2001	Service utilization			X		
Wilkinson et al.	1997	Service utilization			X		
**Senegal (n = 1)**							
Witter et al.	2008	Exemption processes and effects					X
**Tanzania (n = 1)**							
Kruk et al.	2008	Costs of utilization			X		
**Uganda (n = 8)**							
Burnham et al.	2004	Service utilization			X		
Deiniger et al.	2004	Effect of exemption on accessibility and illness			X		
Kajula et al.	2004	Political analysis of exemption	X				
Nabyonga et al.	2005	Service utilization					X
Nabyonga-Orem et al.	2008	Quality of services					X
Pariyo et al.	2009	Service utilization			X		
Xu et al.	2006	Service utilization and catastrophic expenses			X		
Yates et al.	2006	Effects of exemption				X	
**TOTAL**			**3**	**0**	**12**	**2**	**6**

#### *Service delivery*

Many articles mentioned increased service utilization after the implementation of exemption policies, particularly in Madagascar [19], in Uganda for primary care visits [20], and in Ghana and Senegal for institutional deliveries [21-23]. In Uganda, it appeared that the free services could not satisfy the increased service demand, prompting patients who were better-off to use services that were not free [24-26]. This increase in demand therefore put pressure on the health system. This pressure was somewhat alleviated by additional reforms implemented on the supply side (increased budget for drugs, improvements in the stocking system, salary increases) [20]. However, the increase in demand had a negative effect on service quality (non-availability of drugs, longer wait times, decreased motivation among health workers) and, in the end, on malaria control and on users’ confidence in the health system [27]. The decline in service quality could also be attributed to an abrupt change in policy and to underfunding.

#### *Health workforce*

In Uganda, the 2001/2002 administrative data showed that additional salaries were eliminated for auxiliary and technical staff [28]. The absence of this staff who were originally paid by user fees contributed to the human resources crisis [29]. In Uganda, this crisis was seen in the decline in health workers’ morale and in their attitude toward their work [27,28]. Elsewhere, this crisis translated into a heavier workload experienced by health workers [30-33], a feeling of inadequacy among medical personnel [32], and a sense of being overworked and exploited in the face of increasing work demands [30]. Negative effects on practices were reported in South Africa [30]. The loss of income for personnel caused by eliminating user fees could lead doctors to devote themselves more to private practice, especially if the problem of discontinuity in drug supplies were to persist [28].

#### *Health information system*

Only one study, in Ghana, reported that little information was available at the central level on the number and type of deliveries carried out in health centres and on the amount of reimbursements [31].

#### *Medical products, vaccines and technologies*

The experiences indicated an overall shortage of drugs, highlighted in South Africa [30], Kenya [33], and Madagascar [19]. In Uganda, the stock forms showed an increase in the quantity of drugs received after fees were abolished, whereas the actors spoke of problems of availability, particularly for antimalarials, and delivery delays caused by administrative red tape at the district leve [27,29,34]. Patients responded to this shortage by turning to private services to purchase these drugs [27,29]. Some authors reported that health workers perceived the drastic decline in service utilization after seven months of exemption to be a consequence of the shortages in additional drug stocks [28].

#### *Health systems financing*

The major problem is the underfunding of the exemption policies. In Ghana, the funds paid to the districts at the start of the fiscal year were not enough to cover the year’s expenses [31,35]. The policy was underfunded by 34% in 2004 and 73% in 2005. In Uganda, despite an increase in the budgets allocated, the health centres lost income [20,25] and were unable to cover recurring expenses [27]. In Ghana and Kenya, some health centres had resumed charging for services and drugs in order to deal with funding shortfalls [31,33]. Governments sometimes had to gradually reintroduce user fees after they had been abolished [36,37]. In Senegal, health centres increased the fees for certain acts that could still be charged in order to compensate for lost income from those acts that had become free [23].

#### *Leadership and governance*

Most of the countries appeared to have provided clear orientations regarding which services or which populations were the targets of exemption. However, these orientations did not always appear to have been expressed in clear directives, as was seen in South Africa [30]. Ghana [31,32], Kenya [33], and Senegal [23], where it seemed that various actors in the system had been inadequately informed. As well, the exemption policies appeared sometimes to interfere with other health policies and programs, such as community-based health insurance [31]. In Ghana, the difficulty of obtaining accounting reports from managers was an important element for actors in that system [35]. However, the complexity of the reimbursement process and the multiplicity of actors were also factors that impeded a real transparency in the process and made it difficult to assign clear responsibility to actors at different levels in the system (‘no blame’ game).

The various pressures mentioned in the literature are described in Table 3 by country, health system functions, and authors.

**Table 3:** Pressures mentioned in the literature by country, health system functions, and authors (1988 to 2009)

**Health system functions**	**Pressures mentioned in the literature**
***Ghana***	
*Service delivery*	· Increase in utilization [21,31,32,35]
*Health workforce*	· Increase in workload, loss of income [31]
	· Insufficient medical personnel; increase in workload [32]
*Health information*	· No information
*Medical products, vaccines and technologies*	· No information on the number of acts and the amount of reimbursements [31]
*Financing*	· Funding unpredictable, insufficient and discontinuous; problems with reimbursement in cases of referrals [31]
	· Funding unpredictable, insufficient and discontinuous; informal payments; health centres going into deeper debt [35]
*Leadership and governance*	· Lack of information and complexity of funding procedures; poor supervision; problems in assigning responsibilities [31]
	· Lack of information and communication (funding); competition with other interventions; poor supervision; ‘no blame’ game [35]
***Kenya***	
*Service delivery*	· Increase in demand for service [36,37]
*Health workforce*	· Increase in workload [33]
*Health information*	· No information
*Medical products*	· Problems of availability and insufficiency of drugs and kits [33]
*Financing*	· Informal payments [22]
	· Informal payments; loss of income for health centres [33]
	· Insufficient funding; informal payments [36]
*Leadership*	· Poor understanding of the policy; problems in assigning responsibilities [33]
***Madagascar***	
*Service delivery*	· Increase in utilization [19]
*Health workforce*	· No information
*Health information*	· No information
*Medical products*	· Problems of availability of drugs [19]
*Financing*	· No information
*Leadership*	· No information
***South Africa***	
*Service delivery*	· Increase in utilization [29,38-40]
*Health workforce*	· Increase in workload; lack of time for consultations; feeling of being exploited; frustration, etc. [29]
	· Increase in patient/provider ratio [38]
*Health information*	· No information
*Medical products*	· Problems of availability of drugs [29]
*Financing*	· No information
*Leadership*	· Feeling of a lack of recognition among workers; poor planning and communication [29]
***Senegal***	
*Service delivery*	· Increase in utilization [23]
*Health workforce*	· Increase in workload [23]
*Health information*	· No information
*Medical products*	· Delays and under-distribution of consumables [23]
*Financing*	· Informal payments; delays in reimbursements; loss of revenue for the health centres [23]
*Leadership*	· Poor understanding of the policy [23]
***Tanzania***	
*Service delivery*	· No information
*Health workforce*	· No information
*Health information*	· No information
*Medical products*	· No information
*Financing*	· Informal payments [41]
*Leadership*	· No information
***Uganda***	
*Service delivery*	· Increase in utilization [20,24-26,28,34]
	· Increase in utilization; decline in service quality [27]
*Health workforce*	· Lower morale of providers [27,29]
	· Increase in the average number of consultations per provider; negative attitude of providers [22]
*Health information*	· No information
*Medical products*	· Problems of availability of drugs [24,26,27,29,34]
*Financing*	· Insufficient funding [25,34]
	· Difficulties in meeting recurrent expenses; informal payments [27]
*Leadership*	· Interference with other types of interventions [27]

## Discussion

Understanding health systems as complex collections of components that interact in an effort to achieve their objectives offers an opportunity to see the effects of health policies through a new lens [42]. As has been explained by *The Alliance for Health Policy and Systems Research* (AHPSR) [43], every system-wide intervention, such as user fee exemption policies, has an effect on the health system’s different components. This new literature review showed that, in responding to population needs that had up to then not been addressed, particularly because of an unfair funding model, the exemption policies generally created an increase in health services utilization. However, at the same time, this increase produced bottlenecks at different points in the system (Table 4) as was confirmed in a recent supplement of *Health Policy and Planning* on user fee removal in the health sector in low-income countries [44]. That being said, increased utilization was not the only source of pressure on the system. There was a constellation of variables that acted, at the same time, as both causes and consequences of the policies, particularly planning and attendant measures that often proved to be limited [8,44]. There is a risk that, by disrupting the system’s functioning, these interventions will produce the opposite effect from what was intended, that is, a return to low utilization of services. However, given the recent nature of these policies, the timeframe adopted in this review, and the possibility of publication bias, which exposes problems (although often implicitly) more often than successes, we do not yet have enough distance or any evidence that these reverse effects—such as a return to low utilization—actually exist.

**Table 4:** Synthesis of pressures mentioned in the literature (1988 to 2009)

**Health system functions**	**Pressures on the health system**
***Service provision***	Increase in service utilization and in the demand for services
***Health personnel***	Increase in workload, increase in the patient/provider ratio, insufficient medical staff
	Loss of income
	Lack of time for consultations
	Feeling of being exploited, frustrated, overworked
	Negative attitude of providers
	Deterioration in staff morale
***Health information***	Lack of information on the number and type of services carried out in the health centres and on the amount of reimbursements.
***Drugs and vaccines***	Problems of availability of drugs
	Insufficient drugs and kits to meet local needs
	Delays and under-distribution of consumables
***Funding***	Funding unpredictable, insufficient and discontinuous
	Loss of income for health centres and increased debt
	Problems with reimbursements for cases of referrals
	Reverting back to charging for services and drugs
	Insufficient funding
	Service providers having difficulty paying recurrent expenses
	Delays in reimbursements
***Governance and leadership***	Poor planning and communication; poor understanding of the policies
	Inadequate supervision
	“No blame” game and problems in obtaining accounting reports and in assigning responsibilities for acts
	Complexity of funding procedures
	Interference with other health policies and programs

The AHPSR’s observation in 2009 is still relevant with respect to user fee exemption policies: “…*systemic factors and their impacts have hardly been studied*” [43]. The articles included in the present literature review were not aimed at understanding the systemic effect of these policies, but generally focused on a single function of the health system. Two-thirds of the articles addressed only one or two system functions. This perspective adopted by the authors also limits the lessons we can draw from national experiences to support decision-makers involved in developing and implementing such policies. Only a few of the authors attempted to put the results of their studies into perspective in the context of health systems [20,27]. The major lesson to be drawn from this scoping study is thus the need for empirical studies aimed at understanding more clearly, on a nation-wide or district scale, how these policies affect, at one and the same time, *all the functions of the health system*. Now that the positive effects of user fee exemptions on utilization have largely been demonstrated, researchers need to direct their attention toward more systemic questions. This approach fits within the movement that promotes developing and implementing reforms while taking into account the different potential effects on health systems [45]. Addressing these more systemic questions, by looking particularly at implementation, will likely call for new forms of collaboration among researchers, political decision-makers and practitioners [46].

In doing this survey, it was not our intent to report all the available knowledge on these interventions or to discuss the methods used, as this has been done by others already. Our primary aim was to bring to light the elements related to exemption policies that were disruptive to the system and to highlight certain areas where there was a lack of knowledge. The main limitation of our study, aside from its being more narrative than explicative, is therefore that we have not highlighted the successes of these policies, except for the increase in service utilization. Moreover, the elements reported here are only *potential* disruptions and do not necessarily reflect what is actually happening across all systems as a whole. For this reason, we repeat our call for further empirical studies on this matter. Nevertheless, these disruptive elements may also be seen as demonstrations of what might occur if decision-makers, in trying to implement exemption policies, do not ensure the necessary conditions of preparation, planning and attendant measures from a systemic perspective, as is shown in the new empirical studies published in the recent supplement of Health Policy and Planning [44].

## Conclusions

While user fee exemptions for health services have made it possible to address needs that until now had not been met, an analysis of the literature shows the other side of the coin. In fact, in acting on the provision of services to make them accessible to more people, the exemption policies have at the same time disrupted other essential functions of the health systems of the countries involved, which were already, in any case, relatively unstable. If this very relevant user fee exemption policy is to be effective and equitable, it is therefore essential that these interactions be taken into account when formulating exemption policies, in order to limit disruptions and to create the synergy needed for the system to perform optimally.

## Competing interests

The authors declare they have no competing interests.

## Authors' contributions

VR led the study and ER did the literature review. All authors contributed to the interpretation of the results. VR wrote the manuscript with contributions from all authors. All authors read, improved and approved the final manuscript.

## Pre-publication history

The pre-publication history for this paper can be accessed here:

http://www.biomedcentral.com/1471-2458/12/289/prepub
